# Results-Based Financing for Health: A Case Study of Knowledge and Perceptions Among Stakeholders in a Donor-Funded Program in Zambia

**DOI:** 10.9745/GHSP-D-20-00463

**Published:** 2021-12-31

**Authors:** Rachel Bergman, Birger C. Forsberg, Jesper Sundewall

**Affiliations:** a Karolinska Institutet, Stockholm, Sweden.; b Karolinska Institutet, Stockholm, Sweden.; c Lund University and University of KwaZulu-Natal, Malmö, Sweden.

## Abstract

In 2015, the Zambian government and the Swedish International Development Cooperation Agency (Sida) signed an agreement in which Sida committed to funding a program for Reproductive, Maternal, Newborn, Child, Adolescent Health and Nutrition (RMNCAH). The program includes a results-based financing (RBF) model that aims to reward Zambian districts for improved district-wide results on relevant indicators with additional funding. We aimed to describe stakeholders' knowledge of the RBF model and perceptions of the incentive structure during the first 18 months of the program's implementation. This study illuminates the possible pitfalls of implementing an RBF scheme without giving attention to all necessary steps of the process. A qualitative case study was used and included a review of documents, in-depth interviews, and observations. From February–April 2017, we conducted 37 in-depth interviews, representing the views of 12 development partner agencies, government departments, and health facility staff throughout Zambia. We used a qualitative framework analysis. Findings show that the Zambian government and Sida had different perceptions on what levels of the health system RBF will incentivize and that most districts and hospital administrators interviewed were unaware of the indicators that the RBF was part of the RMNCAH program at all. The lack of knowledge about the RBF scheme among respondents suggests the possibility that the model did not ultimately have the necessary preconditions to create an effective incentive structure. These results demonstrate the need for improved communication between stakeholders and the importance of sufficiently planning an RBF model before implementation.

## Abstract (precis)

The lack of a fully developed results-based financing model before implementation of a program in the health sector begins can lead to difficulty in communicating about the program to different actors involved and delay components of implementation.

## Teaser

**Key Messages:**

- The intended incentive structure of results-based financing (RBF) makes it particularly important that program implementers communicate to beneficiaries of a program before implementation begins about the nature of the program and that RBF will be used.
- Improved decision making and communication about the requirements and expectations of an RBF program can enhance stakeholders' knowledge and perceptions of the program, and ultimately make implementation easier.

**Key Findings:**

- The lack of a fully developed RBF model meant the program was more difficult to communicate to the different actors involved.
- The lack of knowledge of the RBF program and the existence of incentives raises doubts about whether the program incentivized actors for the intended targets.

**Key Implications:**

- Program managers should recruit stakeholders from all governments and organizations involved in program financing and implementation to participate in the design of RBF schemes so that they can effectively communicate the structure and indicators to different levels of their respective organizations.
- Program implementers using RBF should have a flexible plan in place for the RBF model before implementation begins, even if the plan might change.

## INTRODUCTION

Results-based financing (RBF) is an umbrella term used to describe any program in which a funder—often a national government or agency, or a foreign development partner—transfers money, material goods, or other incentives to a recipient agent conditional on the recipient achieving predefined output or outcome targets.^1^^–^^3^ An incentive is anything that motivates or encourages the agent to achieve the desired results that are agreed upon with the funder.^4^^,^^5^ Although not distinct from RBF, results-based aid refers to cases in which a bi- or multilateral development partner funds a national government of a partner country and the national government bears the responsibility of delivering results.^2^^,^^6^ Development partners typically see results-based mechanisms as methods to make aid more effective. This position is in line with the 2005 Paris Declaration on Aid Effectiveness, in which participating countries agreed that programs can be managed using a result-based mechanism to improve aid effectiveness.^2^^,^^7^

Oxman and Fretheim provided an overview of systematic reviews analyzing RBF schemes in lower- and middle-income countries (LMICs). They outlined 7 characteristics that can differ between RBF* programs, including the indicators chosen to measure results, the method by which results are measured, the type and magnitude of incentives used, and the level of a government or sector incentivized.^1^ However, their overview, as well as other systematic reviews, shows that there is insufficient evidence to support the concept that RBF leads to better performance, whether it is used to incentivize individuals or entire levels of a sector.^2^^,^^8^ Many studies of RBF programs lack adequate controls that would allow impact to be assessed.^6^ Even when studies have used a randomized control trial design to control for confounders, they have been inconclusive on the impact of RBF.^6^^,^^9^ In fact, evidence from a variety of settings points to the various risks and perverse effects RBF can have.^9^

Still, past RBF schemes have suggested RBF can strengthen health systems and demonstrated key concepts that can help implementers optimize the characteristics of an RBF program for impact.^10^^,^^11^ All stakeholders—both the funders and the recipients—should be involved in the design of the program, and the scheme should be communicated to those who will benefit so that they know the intended results.^1^^,^^6^ Oxman and Freitheim noted specifically that financial rewards and other incentives did not have an impact in countries when intended recipients lacked awareness of the potential rewards.^1^ The choice of indicators used in a program, and choosing ones that the national government knows about and uses, is also critical for the effectiveness of an RBF program.^6^^,^^12^

Although some studies have attempted to analyze the results and impact of RBF, fewer have looked at the challenges involved in actually planning an RBF scheme (i.e., creating and setting one up) especially at the system level rather than individual level.^2^^,^^6^^,^^13^ We aim to fill that gap by providing an understanding of the possible consequences that can arise when the implementation of a program has started but a full plan for implementation of its RBF scheme is not yet developed. In the case of the Reproductive, Maternal, Newborn, Child, Adolescent Health and Nutrition (RMNCAH) program, this resulted in implementers being unable to communicate about the existence of an RBF program and its components, including indicators for which results are measured and the amount of funding recipients can incur for improved results. We examine an RBF scheme designed for the health sector in Zambia, particularly one at this stage of implementation, to use as a case study for assessing the impact of stakeholders' knowledge and perceptions of an RBF model on the model's implementation. The study is illustrative in that it gives an example of how an RBF project is implemented and how it contributes to improved knowledge on how RBF is understood in practice. Additionally, we describe opportunities for improving decision making and in communicating requirements and expectations for an RBF program.

Fewer studies have looked at the challenges involved in planning an RBF scheme, especially at the system level rather than individual level.

## SIDA'S SUPPORT FOR THE RMNCAH PROGRAM

Although the World Bank reclassified Zambia from a low-income country to a lower-middle-income country in 2011, foreign aid continues to be an important part of the Zambian health sector financing as it has for several decades.^14^^,^^15^ The public health sector is structured into national, provincial, district, and community levels.^16^ At the national level, the Ministry of Health (MOH) organizes the overall health sector, and provincial health offices (PHOs) and district health offices (DHOs) provide coordination at the provincial and district levels, respectively. Health service delivery facilities fall into categories at each of these levels: tertiary hospitals at the national level, level 2 general hospitals at the provincial level, level 1 hospitals at the district level, and health posts and health centers at the community level.^16^^,^^17^ Primary health care includes all health services at the district and community levels, which both fall under the supervision of and receive funding from DHOs.^16^

From 2012 to 2014, the World Bank supported a pilot RBF program in Zambian districts that used monetary incentives to motivate individual health care providers to improve and increase the provision of maternal and child health services.^13^ An impact evaluation of the program suggests that districts that received enhanced financing without RBF showed comparable improvement in health care indicators compared to districts that received RBF.^18^

Based on some of the design features of the World Bank's RBF program, in 2015, the Swedish International Development Cooperation Agency (Sida^†^) and the Government of the Republic of Zambia signed an agreement for government-to-government financial support that would last 5 years, from 2016 to 2020, and include fixed and RBF components to improve 5 indicators related to RMNCAH.^19^ Sweden's Appraisal of Intervention, titled “Health support for women, children and youth in Zambia,” provides insight into the country's objectives and intentions regarding this agreement.^20^ The €42.8 million grant included both fixed and variable tranches (VTs), and funds from both would be directed to DHOs for the 22 districts in the southern and eastern provinces of Zambia. Funds could be used at the discretion of districts to support their RMNCAH work, including hiring new staff but excluding bonuses to individual staff members. Despite these decisions about how funds would be disbursed, program documents did not provide any explanation about the theory of change or the incentive structure for disbursements.

The fixed component involved each participating district receiving a portion of a fixed tranche of funds each year (70% of the entire grant) based on the allocation formula the government already uses to disburse money to districts. The Sida funds represented a significant increase in resources at the district level. For many districts, the Sida funds accounted for 20%–30% more on top of their usual government allocations for district-level health care operations, and some districts received an even larger share.

Based on performance according to an RBF model, 30% of the grant money (€12.8 million) was released as a VT. While half of the VT was based on 5 indicators (Table 1) chosen to measure RMNCAH performance (second VT), half was based on a “budget execution indicator” that measured the government's execution of its national health budget by the Ministry of Finance (first VT). The 5 RMNCAH indicators used for the second VT were chosen through discussion between Sida and the Government of the Republic of Zambia, and no process was determined for how they would be changed, if necessary, and how the changes would be communicated to relevant actors. The intention was for there to be 5 annual VTs throughout the project life cycle, with the first disbursement made at the end of 2016. At the time research was conducted in April 2017, a payment had not yet been made, and Sida and the MOH had not finalized the formula that Sida would use to calculate how much of the VT it would release nor how much the MOH would allocate to each district based on their individual performance on the 5 key RMNCAH indicators (Figure).

**FIGURE:**
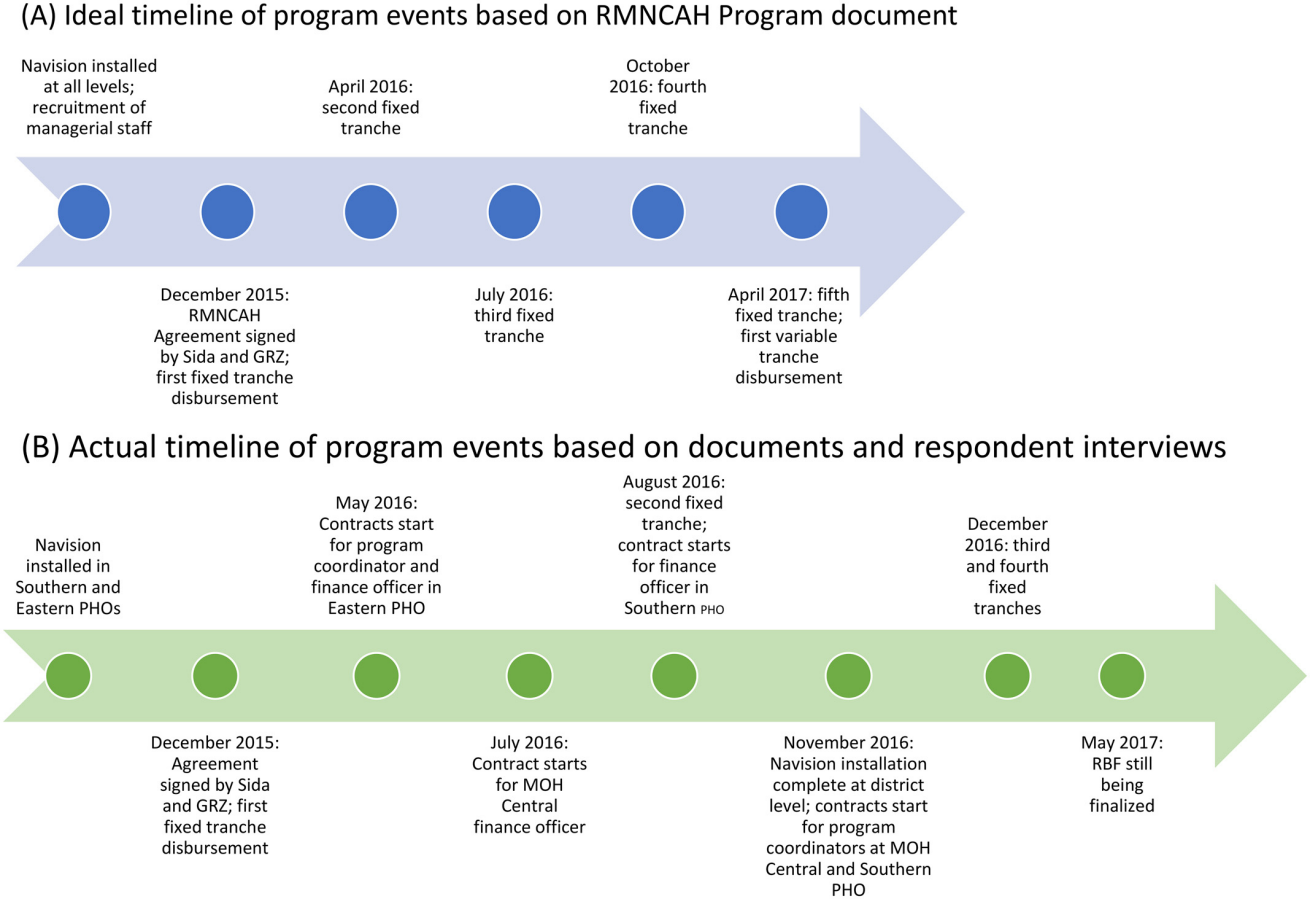
Ideal^a^ vs. Actual^b^ Timelines of Events of RMNCAH Program in Zambia Abbreviations: GRZ, Government of the Republic of Zambia; MOH, Ministry of Health; PHO, provincial health office; RMNCAH, Reproductive, Maternal, Newborn, Child, Adolescent Health, and Nutrition. ^a^Ideal timeline showing that the conditions of the agreement included installment of Navision and recruitment of program management before the first fixed tranche disbursement. It also shows the dates of the first-fourth tranche disbursements according to the provisional schedule in the agreement. ^b^Actual timeline showing when Navision was installed and program management started their contracts. It also shows when disbursements have actually occurred.

**TABLE 1.** Indicators Chosen to Measure RMNCAH Program Performance in Zambia

Original Indicators, 2015	Current Indicators (Changed in June 2016)
Women receiving antenatal care by skilled personnel at least 4 times during pregnancy	Women receiving antenatal care by skilled personnel at least 4 times during pregnancy
Births attended by skilled health personnel	Births attended by skilled health personnel
Newborns receiving postnatal care visits within 2 days of birth	Newborns receiving postnatal care visits within 6 days of birth
Children under 2 receiving the full immunization package	Children under 12 months receiving the full immunization package
Children aged 0–59 months with signs of pneumonia who received antibiotics	Number of women accepting family planning for the first time

Abbreviation: RMNCAH, Reproductive, Maternal, Newborn, Child, Adolescent Health, and Nutrition Program.

Based on performance according to an RBF model, 30% of the grant money is released as a variable tranche.

## METHODS

### Study Design

We used a case study approach, which “allows investigators to focus on a “case” and retain a holistic and real-world perspective”^21^ and study a phenomenon under the assumption that it is not completely distinct from its context. We explored stakeholders' perceptions of implementing the support from Sida for a program for RMNCAH within the broader context of RBF and development assistance for health. The illustrative case study design also allows for a flexible design and holistic understanding using different data sources.^22^ From February to April 2017, we collected data, including reviewing policy documents and other forms of secondary data, conducting in-depth interviews, and collecting observational data.

The main researcher was based in Lusaka, Zambia. That provided access to policy documents, representative stakeholders from the Ministry of Health (MOH), and cooperating partners (CPs)—international agencies and civil society groups that partner with the Zambian government—for conducting interviews and relevant policy meetings regarding the RMNCAH program and health sector overall. Data were also collected from government offices and health facilities in the Southern province and the Eastern province of Zambia.

We defined incentive as something that creates motivation in an individual or among a collective entity to achieve a certain result, which can be specific RBF funding provided to districts through the RMNCAH program or the knowledge that results will be monitored for improvements. We defined program knowledge as including an individual's ability to convey the objective and indicators of the RMNCAH program, as well as his or her understanding that the RMNCAH program included an RBF component.

### Policy Document Review

The study was grounded in an in-depth review of Sida's agreement with the Government of the Republic of Zambia and the Appraisal of Intervention on the RMNCAH program, which provides insight into Sweden's objectives and intentions regarding the agreement. Other policy documents that were reviewed include the MOH's Joint Annual Review Reports from 2008–2012, work plans and budgets for the Eastern and Southern provincial health offices (PHOs), work plans and budgets for selected districts, and the 2016 Zambia Household Health Expenditure and Utilization Survey.

### In-Depth Interviews and Sampling

In-depth, semistructured interviews were used to obtain information and perceptions on the RMNCAH program implementation from key stakeholders at organizations and agencies identified during a preliminary review of relevant policy documents and literature.^23^ We developed an interview guide for each level based on a literature review of the Zambian health sector and information from policy documents. It was piloted and further developed after initial evaluation. Questions were designed to be open-ended to avoid leading the respondents' answers and addressed perceptions of the initial implementation of the RMNCAH program and the understanding of and attitudes regarding the RBF component of the program. New themes that emerged during the interviews, which may not have been foreseen in the initial planning and preparation of the interview guide, were pursued if deemed relevant.

We used purposive sampling to select respondents for interviews based on their ability to provide “intimate knowledge” of RMNCAH, all or parts of the RMNCAH program, or RBF.^24^ In total, we conducted 38 interviews with 37 different respondents from 6 categories: (1) MOH officials (at the central, provincial, and district levels); (2) bilateral development partners (Sida, U.S. Agency for International Development [USAID], and the European Commission); (3) multilateral development partners (the World Bank); (4) nongovernmental organization representatives (Clinton Health Access Initiative [CHAI]); (5) hospital and facility administrators; and (6) health workers. Each category was represented by at least 3 respondents to ensure multiple perspectives from each.

### Attendance at Policy Meetings and Meetings on RBF

The researcher conducted nonparticipatory observations of 3 policy meetings in Lusaka, Chipata, and Eastern Province, as well as 3 RBF program meetings between Sida, MOH officials, and other key stakeholders. Observations were used to understand the process by which the RBF design was created and the perspectives and perceptions that stakeholders involved had about the process and design. These meetings also provided the researchers with a context for the Zambian health sector and the stakeholders involved and insight into how data is collected and analyzed at the district level. Furthermore, this allowed the researchers to identify key informants, such as district health directors and Sida-contracted employees at PHOs as sources for data.

### Data Collection and Analysis

Participants provided verbal consent to the interviewer. The head of the Bilateral Development Cooperation at the Embassy of Sweden, at the time research was conducted, provided written consent for his quotes to be published under his title. A letter from the Permanent Secretary of the MOH endorsing the study was presented to an MOH staff member at the provincial levels. Interviews lasted 1–1.5 hours, and most were conducted within the respondents' place of work. We recorded 23 of the 38 interviews. During all interviews, the researcher took detailed notes, and transcriptions were made after recorded interviews.

Anonymity was ensured to those participants that requested it. The researcher has enhanced anonymity by referring broadly to respondents as “MOH official,” “CP,” “facility administrator,” or “health worker” when appropriate. Program officers for the RMNCAH program, who did not request anonymity, were specifically identified when necessary. When relevant, the level of the health sector at which an MOH official works is specified by referring to them as “MOH Central official,” “PHO official,” or “DHO official.”

A key feature of a qualitative case study is that analysis is conducted throughout the process of data collection.^25^ During interviews and policy meetings, and upon reviewing transcripts or notes, the researcher processed and sifted through data, maintaining a flexible approach that allowed for new ideas and themes to guide further investigation.^25^^,^^26^ Information gathered from these various sources was used to verify collected data. A framework analysis, as described by Ritchie and Spencer, was employed to facilitate a dynamic yet systematic analysis of the interview and observational data, and to ensure that the analysis was grounded in the original accounts of participants and observations made throughout the study.^27^ As an additional analytic technique used throughout the study, predictions made before the start of the research were compared to the emerging data through pattern matching.^21^ Using these techniques, the researchers characterized stakeholders' perceptions regarding which levels of the health system should be incentivized and receive funding, as well as their knowledge of the RBF model and the indicators used to measure performance.

## RESULTS

Results indicate that during the first 18 months of implementation, many respondents responsible for carrying out the RMNCAH program lacked any knowledge that it included an RBF component for disbursing a portion of the grant and had different perceptions on the degree to which DHOs and facilities would be incentivized by the program's VT funding. Many also did not know which specific RMNCAH indicators had been chosen to measure performance. Respondents also revealed misunderstandings regarding the budget execution indicator. At the time of data collection, Sida had not yet dispersed any of the VTs.

### Lack of Knowledge That Program Will Use RBF

Most respondents agreed that funding from the RBF component is meant to target the service delivery points of RMNCAH and that the main purpose is to encourage the districts and facilities to work to improve indicator performance. However, there was less agreement regarding whether individual health workers might feel any extra incentive or motivation from funding received through the RBF component. The Appraisal of Intervention policy document broadly suggests that the RBF is not intended to incentivize individual health workers or facilities. Respondents who believed health workers were aware of the RBF, such as a hospital administrator interviewed, suggested that health workers would feel motivated to contribute and help the facility gain extra resources. In contrast and in line with the development partner's intention, a DHO official stated:

*Individual health workers wouldn't be motivated unless [the RBF] is well explained.*

Most respondents agreed that funding from the RBF component is meant to target the service delivery points of RMNCAH and that the main purpose is to encourage the districts and facilities to work to improve indicator performance.

Table 2 shows the stakeholders that knew the program's RBF component at the time of data collection. Fewer than half of the respondents confirmed they knew that RBF would be used in the RMNCAH program. All MOH Central and PHO officials claimed to know about the use of RBF, but while all MOH Central officials could explain details of the model, not all PHO officials, including RMNCAH program staff, could do the same. The CP who admitted not knowing about the RBF model did not have a role in planning the program.

**TABLE 2.** Respondents' Responses on Knowledge That the RMNCAH Program in Zambia Will Use RBF

Stakeholder Type	Yes	No	Yes/No^a^	Total
CP	5	1	1	7
MOH central official	5	0	0	5
PHO official	4	0	2	6
DHO official	1	3	6	10
Facility staff	1	8	0	9
Total	16	12	9	37

Abbreviations: CP, cooperating partner; DHO, district health office; MOH, Ministry of Health; PHO, provincial health office; RMNCAH, Reproductive, Maternal, Newborn, Child, Adolescent Health, and Nutrition.

a Indicates respondents who had heard of the RMNCAH program's RBF scheme or suggested their familiarity with it but could not or did not provide any detailed information that demonstrated that they had heard of the scheme before the researcher mentioned it.

An MOH central official and 2 PHO officials asserted that the DHOs knew about the program's RBF and the intended monetary incentives directed toward them at the time research was conducted. An RMNCAH program coordinator within the MOH financed by Sida attended the RBF planning sessions with CHAI and Sida. The coordinator collaborated on the design and was responsible for communicating about the program and RBF scheme to the PHOs and DHOs through 1-on-1 meetings and policy meetings. One of 10 respondents interviewed at DHOs could provide any information that confirmed that they knew it would be used. The 3 respondents that admitted that they did not know that the program would use RBF were all district health directors (i.e., the top officials responsible for health at the district level).

Additionally, several MOH officials expressed the perception that while the DHOs would feel motivated by receiving extra funds, the VT based on RMNCAH performance wouldn't serve as an “incentive” for them even though it could expand funding:

*The DHO doesn't have much incentive because we won't share it among ourselves…it's just extra resources to run programs. —MOH official*

“Facility staff” refers to hospital administrators and health workers at both hospitals and clinics. Two MOH officials and a hospital administrator stated that facilities and health workers were aware of the RBF, yet of 6 administrators and practitioners interviewed, the only respondent at a facility that knew about the RBF was the hospital administrator.

### Lack of Knowledge Regarding Indicators for the Second VT

The development partner's vision for the principles that should guide the RBF component of its support to Zambia for the RMNCAH program are outlined in the Appraisal of Intervention:

*When linking finance to performance it is very important that the indicators are relevant and measurable and that the measurements can be done in an objective and indisputable way.*

This policy document also states that the 5 RMNCAH indicators chosen to measure performance for the second VT were chosen from 29 coverage indicators “that can monitor real-time change” in data and are all regularly recorded in the national health management information system (HMIS). However, respondent interviews revealed that 3 of the 5 RMNCAH indicators initially chosen are not tracked in the government's HMIS, a system for collecting data from various levels of the health sector.

According to an MOH central official, the indicators were changed to indicators that are included in regular data collection in facilities in June 2016, halfway through the first performance year, when the MOH wanted to start reporting indicator performance. At that point, facilities and districts should have started measuring the indicators for the first 2 quarters of the year.

Most respondents directly involved with the RMNCAH program or Zambian health system—Sida and USAID staff, MOH officials, and health care workers or administrators—were asked if they could list the performance indicators used (Table 3). Fewer than half of respondents could name the indicators, and 2 named the initially chosen indicators that have since been changed (“cite the old ones”). All CPs, 3 of 5 MOH Central officials, and half of the PHO officials interviewed could name the specific indicators. At DHOs, where indicator performance is evaluated, only 1 of 10 respondents could name the correct indicators, which was fewer than any other stakeholder type. Two of the 5 respondents at the facilities named the correct indicators.

**TABLE 3.** Respondent's Knowledge of RBF Performance Indicators^a^ for RMNCAH Program in Zambia, N=30

Stakeholder Type	Yes^b^	No^c^	Cite June 2016 Indicators	Total
CP	4	0	0	4
MOH central official	3	2	0	5
PHO official	3	3	0	6
DHO official	1	7	2	10
Facility staff	2	3	0	5

Abbreviations: CP, cooperating partner; DHO, district health office; MOH, Ministry of Health; PHO, provincial health office; RBF, results-based financing; RMNCAH, Reproductive, Maternal, Newborn, Child, Adolescent Health and Nutrition.

a Some individuals included in the summarized responses looked up the indicators on their computers. However, all of them did not respond with the correct indicators, and thus this should not affect analysis.

b Individuals who responded with at least 4 of 5 of the new key indicators are included in the “yes” column.

c Some individuals included in the “no” column named the indicators in the Program Document for the goal to “reduce maternal, newborn and child mortality and malnutrition” but did not name the specific indicators used to measure results for RBF.

One PHO official who could name the performance indicators ultimately used in 2016 and knew that 3 had been changed expressed uncertainty about which indicators would be used for 2017 stated:

*I hope that there's been revision of these indicators. They've been replaced for the annual report in 2016, but I don't know if they'll stay [for the rest of the program].*

The head of the Bilateral Development Cooperation at the Embassy of Sweden expressed the perspective that it was not a problem that most respondents involved in the program at the district and facility levels did not know the 5 key indicators used for reporting in 2016:

*[People] should [know]. But on the other hand, the incentive shouldn't be to achieve the indicators. The incentive should be to achieve certain results…I'm not saying it's not necessary [for people to know] – I'm sure there could have been advantages with that. But it's potentially also problematic – the reason we have many indicators is also that you shouldn't change your work in order to achieve 1 indicator.*

### Lack of Plans for VT Disbursement

Before starting the program, CPs and MOH Central officials had not established a method for calculating how much of the VT each district would receive. At the time research was conducted, over a year into the program, meetings were held to determine the formula that would be used to calculate disbursement.

Representatives from Sida, MOH Central, and CHAI took part in initial meetings about the disbursement formula, and USAID representatives joined for future meetings. Discussions revolved around how the program would measure improvement on the chosen indicators and the proportion of the VT a district would receive based on different levels of performance.^1^ A representative from CHAI presented different models for disbursement, suggesting that district performance could be compared based on raw changes in measurements of indicators or changes in measurements proportional to population.

During interviews separate from these meetings, Sida staff reasoned that it was an advantage not to have this aspect of the RBF model set before the start of the program because it allowed for those involved with the design to factor in lessons learned from the first year of the program's implementation.

In answering a question about why the RMNCAH program was started even though the MOH had not implemented certain technical requirements that Sida had requested in the terms of the program, the head of the Bilateral Development Cooperation at the Embassy of Sweden shed light on why the RBF was launched without a complete design:

*We wanted the program to start, but we had also waited for years. And every year we wait, we either have to do nothing, which has consequences, or other things that we didn't necessarily fancy, like working through NGOs or other contractors outside the government…so of course we wanted to have everything in perfect order before we started. On the other hand, that's not going to happen, so we had to start something.*

## DISCUSSION

This study reviewed the establishment of a program in the health sector in which RBF played a significant role 18 months after it started. We contacted a broad set of stakeholders to assess if there was a difference in knowledge between people working in high and low levels of the health sector and whether the knowledge cooperating partners had corresponded to actors within the high or low levels of the sector. We found that actors from top to bottom of the health system did not have the same perceptions or level of knowledge regarding the program's RBF scheme. Beyond gaps in knowledge, stakeholders also had perceptions about the RBF component that did not align with each other nor with the intended purpose of using the RBF tool as outlined in project documents. Here, we discuss the underlying reasons for these gaps in knowledge and differing perceptions, the possible impacts they have on the successful implementation of RBF in a health system, and potential ways to improve knowledge and alignment in perceptions in the future.

We found that actors from top to bottom of the health system did not have the same perceptions or level of knowledge regarding the program's RBF scheme.

Although data on the 5 selected indicators were collected in the first year of the program, details about the RBF model had not been communicated throughout the health system. Fewer than half of the 37 respondents expressed a complete lack of knowledge regarding the use of RBF, and almost all the respondents at the district level did not know RBF will be used or could not provide any details or information about how the model will work.

The purpose of the RBF model, as described in both documents and discussions with CPs and MOH officials, is to incentivize better performance and service delivery, although stakeholders had differing perceptions on whether money should go to the DHOs or the facilities. By definition, an incentive provides individuals or groups with motivation for a certain course of action or behavior.^5^ Thus, for an RBF model to create an effective incentive structure, a minimum precondition is that those targeted by the model know that the model and the incentive exist. The RMNCAH RBF model was intended to target individuals at the district and facility levels, yet interviews with them suggest knowledge of the model was very limited. In fact, this level of the system had the lowest fraction of individuals that were aware of the scheme. This raises questions about how effectively they are incentivized by RBF.

Importantly, the RBF model is also meant to incentivize the health workers and health facility staff. Lack of communication may be the reason stakeholders had different perceptions on who should be incentivized, which in turn can have implications on the program outcomes. If individuals at these levels had known about RBF from the start of the grant period, they may have felt motivated to ensure that their performance was measured based on accurate data from regularly reported indicators and may have realized the issue earlier during the implementation process.

Beyond stakeholders' doubts that the incentives are effective, Paul et al. noted that performance-based financing programs can weaken health systems in cases when recipients do not have a clear understanding of how and why they get RBF bonuses.^9^ Subnational health officials and health workers play key roles in implementing any program on the ground and can counter challenges that arise throughout implementation when the design and goals are communicated to them.^28^ When individuals on the ground lack knowledge about RBF, those most influenced by the policy cannot offer feedback and help improve it. For instance, CPs and MOH central officials did not notice for several months that the RMNCAH performance indicators they chose are not all included in the HMIS. This implied that the indicators selected were not sufficiently adapted to the Zambian context. A factor in this may have been that the CPs had special requests that the MOH felt obliged to comply with even if it meant putting new requirements on the established system. Health workers and staff at facilities and DHOs are very familiar with the indicators tracked in HMIS as it is part of their job to collect and input data into the system.

When individuals on the ground lack knowledge about RBF, those most influenced by the policy cannot offer feedback and help improve it.

The low numbers of individuals at the district and facility levels who could name the performance indicators and that the initial indicators were not all tracked in HMIS also demonstrate that the indicators have not been internalized in the health system. Literature suggests that a national government should be fully aware of the indicators used and feel a sense of power over them to optimize the impact of RBF.^6^^,^^29^ Yet, according to the United Nations Development Program, studies often indicate that the biggest challenge to performance management is choosing indicators that will satisfy the objectives of all stakeholders at various levels within a given public sector.^1^ Thus, regardless of whether or not individuals in the DHOs and facilities generally know if an RBF scheme will be used, the lack of awareness regarding the indicators the model uses could affect the eventual impact of the scheme.

The head of Bilateral Development Cooperation at the Swedish Embassy in Lusaka indicated the perception that although people should know what indicators are used, they do not need this knowledge for the RBF to work. Sida wants the health sector to achieve improvements in RMNCAH overall rather than just on specific indicators. The perception of the head of the Bilateral Development Cooperation reflects a worry that RBF recipients will neglect unrewarded activities that are not measured and distort other health outcomes.^3^ This type of “perverse incentive”—an incentive that can unintentionally lead to undesired results that undermine the aims of a program—has been noted in past RBF studies.^1^^,^^2^^,^^30^ In an analysis of an RBF scheme implemented by the Gavi, the Vaccine Alliance, Heaton and Keith suggested as a solution to this perverse incentive that programs use performance indicators based on components of health systems strengthening instead of specific health indicators.^3^^,^^31^ Still, while the possibility of distortion is a concern, the use of indicators to measure results is an inherent characteristic of an RBF scheme, and thus respondents' lack of knowledge about them implies a lack of communication within the health system.^1^

The use of indicators to measure results is an inherent characteristic of an RBF scheme, and thus respondents' lack of knowledge about them implies a lack of communication within the health system.

Sida staff reasoned that the lack of a finalized RBF formula that would be used to calculate dispersals of the VT was advantageous. This standpoint is supported by frameworks that promote concepts of iterative learning to design development programs. In their problem-driven iterative adaptation approach to development interventions, Andrews et al. (2012) proposed that a key element of system reform is active learning through experimentation of design and implementation. In low-resource settings like Zambia, implementing interventions should allow for the context to shape the design on a continuous basis and incorporate lessons learned from what works and what does not work on the ground.^32^ When the approach has been put into practice, the use of iterative learning has allowed national governments to work through challenges that arise in projects driven by development partners.^33^

Still, to use a process of iterative learning, a program must be planned at least to the extent that it can be implemented and experimented in actual practice. The RMNCAH program started with an incomplete RBF design. Sida and the MOH waited for feedback about the chosen indicators and information from the lower levels of the health system that might help them develop the RBF component's design, but the lower levels of the system lacked knowledge about the program that would allow them to provide feedback because they were waiting for instructions about the RBF component and what the program actually included. Both waited for communication and additional knowledge about the program from each other, which meant that initial implementation and experimentation never occurred and information could not be gathered about the functionality of RBF in the Zambian context. Communicating about an initial plan, even if it will be revised, necessitates creating a process of information dissemination that can help gather feedback about the initial design from participants and can help clarify channels of communication for disseminating any changes to the plan or additional information in the future.

Previous literature^6^^,^^34^ on strategic planning and RBF schemes asserts that planning, in general, is necessary for assessing results and impact and monitoring any progress that occurs. Thus, a continued lack of planning may affect the efficient exchange of knowledge and comprehensive assessment of the program's results moving forward.

### Limitations

While this study achieved its aims, it did suffer from certain limitations. Due to a limit of time and resources, the researcher could not interview more individuals in remote districts, which could have provided a broader range of perceptions regarding RBF and the intended incentive structure, as well as a larger pool of respondents to better understand communication between various levels of the health system. The researcher could not contact some of the stakeholders in the RMNCAH agreement and implementation that stakeholders mentioned in interviews. It was also difficult to return to respondents for a second interview, so some information gathered in the process of data collection could not be triangulated by all types of stakeholders. The limit of times and resources also meant that the research was designed to only focus on the initial phase of implementation and did not follow the RMNCAH program through its completion. Ideally, further studies would be conducted to look at implementation after a longer period has passed since the program's initiation. Additionally, during fieldwork in Southern province, the researcher was largely subject to supervision from the PHO. A driver was provided for transportation and PHO staff introduced the researcher to relevant staff at the DHOs and facilities. The researcher accepted this supervision as a way of adhering to MOH protocol and for convenience in accessing respondents. These introductions, however, may have altered the respondents' perceptions of the researcher and limited the scope of possible informants sought by the researcher.

## CONCLUSION

This study illuminates important considerations for planning and implementing an RBF scheme throughout an entire sector. Stakeholders interviewed displayed limited knowledge about the RBF model, the results that would be rewarded, and how the rewards would be calculated. Although an RBF scheme might benefit from being iterated throughout the implementation period, it is recommended that a flexible plan is in place for a program during the initial phase of implementation so that all actors have at least basic knowledge and understanding from the onset on its intended results and incentive structure. We recommend that all relevant stakeholders from each government or organization participate in the design of the RBF scheme so that they can effectively communicate the structure and indicators to the different levels of their respective organizations. While these recommendations apply to RBF schemes, preparation and a shared understanding of programmatic details among stakeholders is important for any reforms to the health sector.

A follow-up to this study could examine if the challenges experienced during implementation impacted performance and the ability of Zambian districts to make improvements on the chosen RMNCAH indicators. More broadly, research could examine the minimum knowledge RBF recipients need about an RBF model implemented in LMICs to create an effective incentive structure and determine the preconditions for a viable scheme.

## Footnotes

* RBF schemes can take various forms depending on many characteristics, including who is funded and targeted by the incentives. For instance, in the health sector, a development partner can fund a recipient government or target individual health care providers. A discussion of the full range of varying RBF characteristics is outside of the scope of this article. See Oxman and Fretheim for more information.

† In Zambia, Sida is represented by the Embassy of Sweden. Although the Embassy is responsible for the appraisal and follow-up of any financial contributions, Sida provides the funding and is the name most often used by stakeholders of an agreement to identify the Swedish government as a financial development partner. Thus, we use Sida to refer to Sweden's role as a development partner in Zambia.

## Notes

**Peer Reviewed**
